# Inpatient Coronary Angiography and Revascularisation following Non-ST-Elevation Acute Coronary Syndrome in Patients with Renal Impairment: A Cohort Study Using the Myocardial Ischaemia National Audit Project

**DOI:** 10.1371/journal.pone.0099925

**Published:** 2014-06-17

**Authors:** Catriona Shaw, Dorothea Nitsch, Retha Steenkamp, Cornelia Junghans, Sapna Shah, Donal O’Donoghue, Damian Fogarty, Clive Weston, Claire C. Sharpe

**Affiliations:** 1 UK Renal Registry, Southmead Hospital, Bristol, United Kingdom; 2 Department of Renal Sciences, Division of Transplantation Immunology and Mucosal Biology, Kings College London, London, United Kingdom; 3 London School of Hygiene and Tropical Medicine, London, United Kingdom; 4 Department of Epidemiology and Public Health, University College London, London, United Kingdom; 5 Department of Renal Medicine, Kings College Hospital, London, United Kingdom; 6 Department of Renal Medicine, Salford Royal NHS Foundation Trust, Salford, United Kingdom; 7 Department of Renal Medicine, Belfast Health and Social Care Trust, Belfast, Northern Ireland, United Kingdom; 8 Myocardial Ischaemia National Audit Project, College of Medicine, Swansea University, Swansea, Wales, United Kingdom; University of Miami Miller School of Medicine, United States of America

## Abstract

**Background:**

International guidelines support an early invasive management strategy (including early coronary angiography and revascularisation) for non-ST-elevation acute coronary syndrome (NSTE-ACS) in patients with renal impairment. However, evidence from outside the UK suggests that this approach is underutilised. We aimed to describe practice within the NHS, and to determine whether the severity of renal dysfunction influenced the provision of angiography and modified the association between early revascularisation and survival.

**Methods:**

We performed a cohort study, using multivariable logistic regression and propensity score analyses, of data from the Myocardial Ischaemia National Audit Project for patients presenting with NSTE-ACS to English or Welsh hospitals between 2008 and 2010.

**Findings:**

Of 35 881 patients diagnosed with NSTE-ACS, eGFR of <60 ml/minute/1.73 m^2^ was present in 15 680 (43.7%). There was a stepwise decline in the odds of undergoing inpatient angiography with worsening renal dysfunction. Compared with an eGFR>90 ml/minute/1.73 m^2^, patients with an eGFR between 45–59 ml/minute/1.73 m^2^ were 33% less likely to undergo angiography (adjusted OR 0.67, 95% CI 0.55–0.81); those with an eGFR<30/minute/1.73 m^2^ had a 64% reduction in odds of undergoing angiography (adjusted OR 0.36, 95%CI 0.29–0.43). Of 16 646 patients who had inpatient coronary angiography, 58.5% underwent inpatient revascularisation. After adjusting for co-variables, inpatient revascularisation was associated with approximately a 30% reduction in death within 1 year compared with those managed medically after coronary angiography (adjusted OR 0.66, 95%CI 0.57–0.77), with no evidence of modification by renal function (p interaction = 0.744).

**Interpretation:**

Early revascularisation may offer a similar survival benefit in patients with and without renal dysfunction, yet renal impairment is an important determinant of the provision of coronary angiography following NSTE-ACS. A randomised controlled trial is needed to evaluate the efficacy of an early invasive approach in patients with severe renal dysfunction to ensure that all patients who may benefit are offered this treatment option.

## Introduction

Thirty to forty percent of patients presenting with NSTE-ACS have renal impairment [1]. Compared with patients with preserved renal function those with impairment have a 2–5 fold greater risk of death after NSTE-ACS; those with most severe renal impairment being at highest risk [2]. The projected annual cost to the National Health Service (NHS) of additional cardiovascular events occurring in patients with chronic kidney disease (12 000 myocardial infarctions and 7 000 strokes per year) is £174–178 million [3].

Generally an ‘early invasive’ approach after NSTE-ACS – characterised by routine coronary angiography, followed where possible by early percutaneous or surgical revascularisation – has been demonstrated to improve patient survival [4]. Yet patients with renal impairment were under-represented in the clinical trials that showed this benefit [5]. Current European and American guidelines advise early angiography after NSTE-ACS *irrespective* of renal function [6], [7]. However, several reports from outside the UK suggest that patients with renal dysfunction are significantly less likely to undergo angiography or subsequent revascularisation [1], [8]–[10]. Reasons for this discrepancy, between guidelines and practice, are likely to be complex. Remaining uncertainty as to whether renal dysfunction negates the benefit associated with early revascularisation may contribute.

We used data from the Myocardial Ischaemia National Audit Project (MINAP) to describe and quantify use of an early invasive approach after NSTE-ACS in those with normal and those with impaired renal function in NHS clinical practice. We investigated the association between inpatient coronary angiography and death. Furthermore, for patients undergoing inpatient angiography, we investigated whether renal dysfunction at the time of presentation modified the association between revascularisation and death within 1 year.

## Methods

### Study Population

Care of patients presenting with ACS to all acute NHS hospitals in England and Wales are monitored through MINAP [11]–[13]. Briefly, each patient entry contains prospectively collected information on aspects of diagnosis, investigation and management. The project uses highly secure electronic systems of data entry and transmission, and allows linkage with the NHS Central Register for mortality tracking. Assurance of data quality involves continual monitoring of key fields and an annual validation exercise. MINAP is supported by the British Cardiovascular Society under the auspices of the National Institute for Cardiovascular Outcomes Research (NICOR) and is commissioned and funded by the Healthcare Quality Improvement Partnership.

Anonymised data from an adult population with a diagnosis of NSTE-ACS admitted to hospital between 1^st^ Jan 2008 and 31^st^ March 2010 were used. The diagnosis of NSTE-ACS was made by the local clinician using their judgement of presenting symptoms and requiring elevated blood troponin concentration, with or without electrocardiographic changes consistent with ischaemia. Patients with ST elevation were excluded from this analysis.

### Study Exposures

The first single serum creatinine (µmol/l) within 24 hours of admission was used to estimate the glomerular filtration rate (eGFR) in ml/minute/1.73 m^2^ using the equation developed by the Chronic Kidney Disease Epidemiology Collaboration (CKD EPI) [14]. All creatinine values were assumed not to have been calibrated by isotope dilution mass spectrometry and therefore were multiplied by a 0.95 standardisation factor. Renal function was initially categorised as eGFR>90 ml/minute/1.73 m^2^, eGFR 60–90 ml/minute/1.73 m^2^, eGFR 45–59 ml/minute/1.73 m^2^, eGFR 30–44 ml/minute/1.73 m^2^, eGFR 15–29 ml/minute/1.73 m^2^ and <15 ml/minute/1.73 m^2^ for the descriptive analysis [15]. As relatively low numbers of patients with an eGFR 15–29 ml/minute/1.73 m^2^ and <15 ml/minute/1.73 m^2^ underwent inpatient coronary angiography or inpatient revascularisation the two eGFR categories were combined for subsequent analyses (eGFR<30 ml/minute/1.73 m^2^].

Inpatient revascularisation was defined as inpatient percutaneous coronary intervention (PCI) or coronary artery bypass grafting (CABG). Patients were categorised as medically managed following inpatient coronary angiography if i) PCI or CABG was planned after discharge, or ii) the patient refused such interventions, or iii) the procedures were neither planned nor performed during the index admission.

### Study Outcomes

The primary study outcomes were performance of inpatient coronary angiography – dichotomised as performed or not performed – and all-cause death within one year of presentation. Patients who died on the day of admission were excluded from analyses.

### Confounder Variables

Demographic factors included age (10 year categories), sex, ethnicity, hospital of admission and self- reported smoking status. The Index of Multiple Deprivation was included. This index reflects information on the seven domains of income: employment; health and disability; education, skills and training; barriers to housing and services; living environment; and crime [16]. Co-morbidities included a history of hypertension, previous angina, previous myocardial infarction, hyperlipidaemia, peripheral vascular disease, cerebrovascular disease, chronic obstructive airways disease, congestive cardiac failure, diabetes mellitus, previous PCI and previous CABG. Haemoglobin (g/dl) recorded within 24 hours of admission and peak troponin were also used.

We lacked direct measurements of left ventricular function. Surrogates for reduced function included a history of congestive cardiac failure or previous myocardial infarction. Systolic blood pressure (SBP) and heart rate at the time of admission are validated prognostic markers in ACS and thought to be representative of the degree of acute left ventricular dysfunction [17]. The first SBP (mmHg) recorded after admission to hospital was used. If the patient presented with a treatable tachyarrhythmia, the first stable SBP after treatment was used. The heart rate (beats/minute) was recorded from the first ECG after admission to hospital, whilst in a stable cardiac rhythm. The ECG appearances at presentation were included (normal, left bundle branch block, ST segment depression, T wave changes only, other abnormality).

### Statistical Analyses

All statistical analyses were done using STATA version 11.2.

Confounder exposure associations were cross-tabulated both in the full study population and in the subgroup of patients undergoing inpatient coronary angiography. The frequency and proportions of missing data within each variable were tabulated and distributions of population characteristics for participants included in the complete case analysis were compared with individuals who were excluded due to incomplete data on the *a priori* variables.

Univariable and then multivariable logistic regression models adjusted for all study covariables were used to estimate the odds ratio for the association between eGFR category and undergoing inpatient coronary angiography. Robust standard errors were used to account for clustering at hospital level.

Logistic regression models were also used to assess the association between inpatient coronary angiography and all-cause death. As it was expected that in some cases those that did not undergo inpatient angiography would vary substantially in their baseline characteristics compared with those that did, a propensity score was estimated to help ensure adequate overlap between the distributions of confounders in the two treatment groups [18]. The analysis was repeated restricting to a sub group of the cohort with improved balance in baseline co-variables. The propensity score was the conditional probability that an individual had inpatient coronary angiography and was obtained for each individual by fitting a logistic regression model with outcome inpatient coronary angiography with all the pre-specified co-variables included. All of the pre-specified co-variables were considered *a priori* confounders.

Diagnostic coronary angiography is a pre-requisite for being considered for revascularisation. The analysis to evaluate whether renal dysfunction modified patient survival after inpatient coronary revascularisation compared with medical management was therefore limited to individuals who underwent inpatient coronary angiography. Again a propensity score was estimated to help ensure adequate overlap between the distributions of confounders in the two treatment groups. The propensity score was the conditional probability that an individual had inpatient revascularisation, and was obtained for each individual by fitting a logistic regression model with outcome inpatient revascularisation with all the pre-specified co-variables. After estimation of the conditional propensity score one patient from the medically managed group was excluded as they could not be matched due to a very low propensity score. Improved balance in the distribution in the co-variables between the two treatment groups was achieved (Appendix S1). Multivariable logistic regression analysis was subsequently carried out using robust standard errors with outcome death or alive within one year. Evidence of effect modification between eGFR category and inpatient revascularisation or medical management on the odds of death within one year was tested (Wald test). Evidence of effect modification between gender and inpatient revascularisation or medical management was also tested [19]. The interaction terms were maintained in the model at a threshold of p<0.01. Results are presented as multivariable adjusted odds ratios with 95% confidence intervals.

### Sensitivity Analyses

Logistic regression with the propensity score included as the single co-variable was conducted. Robust standard errors and bootstrapping methods (50 repetitions) were used.

To evaluate possible bias introduced by patients who died early after admission to hospital the analyses were repeated using a cohort limited to individuals who survived five days or more.

Secondary preventative medications including aspirin, clopidogrel, ACE inhibitors, beta-blockers and statins have been shown to influence outcome after NSTE-ACS [20]–[23]. Whether these medications were prescribed at time of discharge was included in the multivariable model evaluating the association between inpatient revascularisation and death within one year.

Sensitivity analysis using datasets derived using multiple imputation was also conducted [24].

### Ethical Approval

Ethics committee (11/L0/0246), Kings College Hospital Research and Development (KCH11-081), and MINAP Academic Group approvals were obtained prior to commencement of the analysis.

## Results

### Renal Impairment at the Time of Presentation with NSTE-ACS and Subsequent Inpatient Coronary Angiography

**Figure 1:**
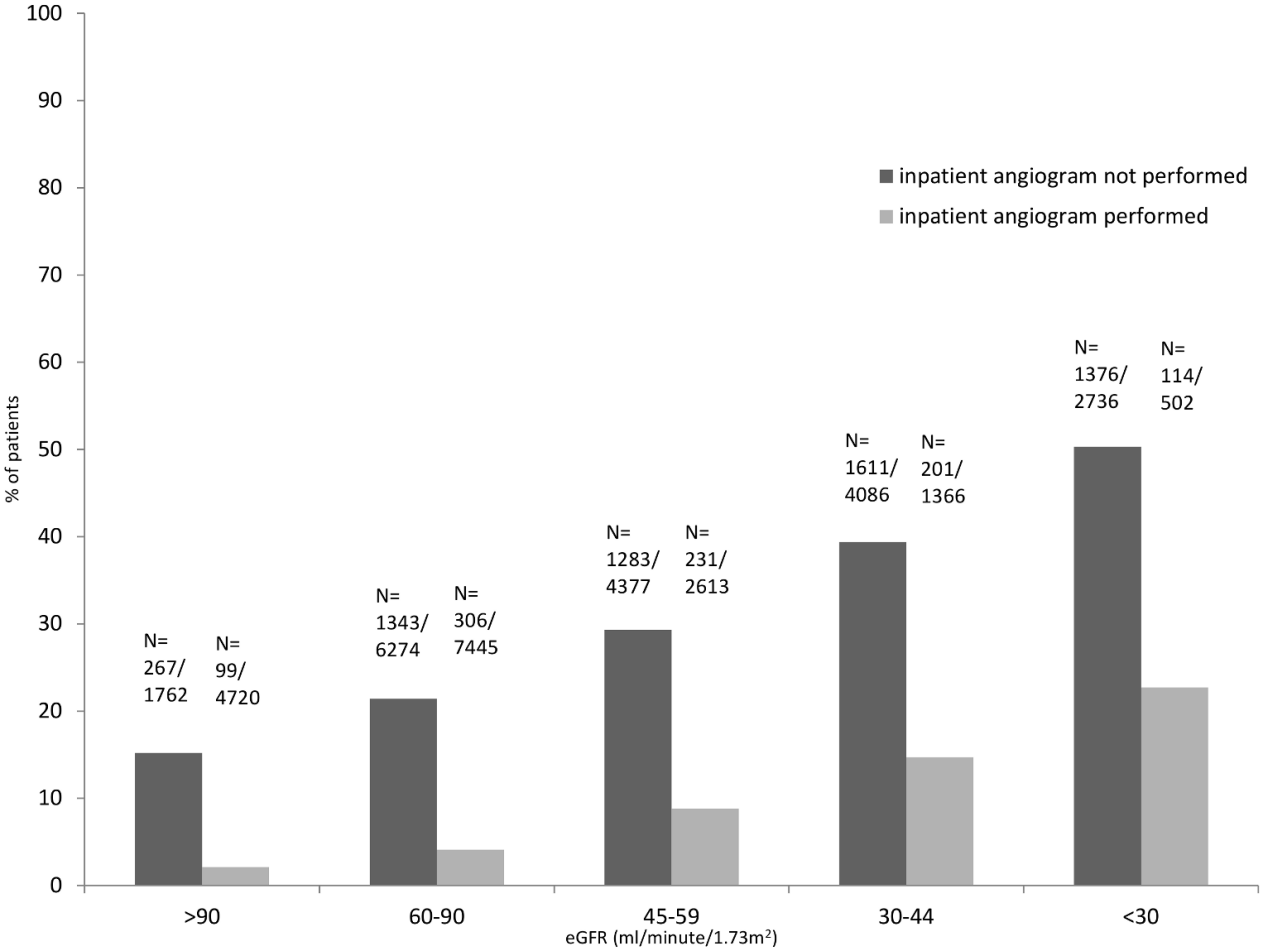
Percentage of patients that died within 1 year after non-ST-elevation acute coronary syndrome. Percentage of patients that died within 1 year after non-ST-elevation acute coronary syndrome stratified by category of estimated glomerular filtration rate at the time of presentation and whether inpatient coronary angiography was performed. *Abbreviations eGFR = estimated glomerular filtration rate; *this analysis included 35 881 patients presenting with NSTE-ACS.

GFR could not be estimated for 18.2% (16 632/91 342) due to missing data on creatinine, gender, age or ethnicity (Appendix S2). Data was missing regarding coronary angiography in 4.5%, management strategy (inpatient revascularisation or medical management) in 18.3% and for mortality in <1% of patients. 15.8% of individuals excluded from the complete case analysis due to incomplete data died compared with 19.0% of those included (Appendix S3). Complete data on all co-variables was available in 35 881 cases. Approximately 40% (n = 15 680/35 881) had an eGFR<60 ml/minute/1.73 m^2^, and 9.0% (n = 3 238) an eGFR<30 ml/minute/1.73 m^2^ (Table 1). The median age was 75 years, and 22 425 (62.5%) were male. Individuals with impaired renal function tended to be older, with a higher co-morbid profile, and more likely to die within 1 year (Table 1, Figure 1).

**Table 1:** Selected covariates stratified by eGFR category at time of presentation in 35 881 adults presenting with non-ST-elevation acute coronary syndrome (all data is presented as numbers with column percentage unless otherwise stated).

	eGFR (ml/minute/1.73 m^2^)					
	>90	60–90	45–59	30–44	15–29	<15
	N = 6 482	N = 13 719	N = 6 990	N = 5 452	N = 2 665	N = 573
**Demographic**						
Male gender	4781(73.8)	9223(67.2)	4010(57.4)	2749(50.4)	1326(49.8)	336(58.6)
Age, median (IQR)	58(50–66)	72(63–80)	79(72–85)	83(77–88)	84(78–88)	80(73–86)
**Past Medical History**						
Hypertension	2680(41.4)	7089(51.7)	4161(59.5)	3375(61.9)	1671(62.7)	387(67.5)
Stroke	333(5.1)	1276(9.3)	950(13.6)	883(16.2)	439(16.5)	107(18.7)
PVD	241(3.7)	591(4.3)	442 (6.3)	390(7.2)	235(8.8)	71(12.4)
Treated hyperlipidaemia	2243(34.6)	4805(35.0)	2449(35.0)	1866(34.2)	854(32.1)	177(30.9)
CCF	128(2.0)	723(5.3)	688(9.8)	917(16.8)	584(21.9)	106(18.5)
Previous MI	1382(21.3)	3964(28.9)	2662(38.1)	2394(43.9)	1318(49.5)	256(44.7)
Previous PCI	787(12.1)	1488(10.9)	727(10.4)	549(10.1)	227(8.5)	52(9.1)
Previous CABG	362(5.6)	1121(8.2)	695(9.9)	536(9.8)	256(9.6)	57(10.0)
Diabetes Mellitus	1150(17.7)	2727(19.9)	1748(25.0)	1735 (31.8)	965(36.2)	235(41.0)
Current smoker	2820(43.5)	2948(21.5)	950(13.6)	520(9.5)	237(8.9)	59(10.3)
**Diagnostics**						
Haemoglobin (g/dl), median (IQR)	14.2(13.0–15.2)	13.8(12.4–15.0)	13.0(11.7–14.1)	12.0(10.9–13.5)	11.3(10.0–12.6)	10.6(9.5–12.0)
Peak Troponin, median(IQR)	0.7(0.2–3.1)	0.7(0.2–3.3)	0.8(0.2–3.9)	0.9(0.2–4.1)	1.2(0.3–5.3)	1.7(0.4–8.2)
Systolic blood pressure (mmHg), mean (sd)	143(26)	144(28)	142(29)	140(30)	135(31)	137(33)
Heart rate (beats/min), median (IQR)	77(66–90)	78(66–93)	82(69–99)	85(71–101)	85(71–100)	86 (71–100)
IP Coronary angiography	4720(72.8)	7445(54.3)	2613(37.4)	1366(25.1)	416(15.6)	86(15.0)
IP revascularisation	2992(46.2)	4422(32.2)	1370(19.6)	697(12.8)	205(7.7)	46(8.0)
IP PCI	2758(42.5)	3977(29.0)	1208(17.3)	609(11.2)	183(6.9)	44(7.7)
IP CABG	234(3.6)	445(3.2)	162(2.3)	88(1.6)	22(0.8)	2(0.3)

Abbreviations: IMD score = score of deprivation; PVD = peripheral vascular disease; CCF = congestive cardiac failure; MI = myocardial infarction; PCI = percutaneous coronary intervention; CABG = coronary artery bypass graft; eGFR = estimated glomerular filtration rate; IP - inpatient; IQR = interquartile range; sd = standard deviation.

Inpatient coronary angiography was performed in 16 646 (46.4%) of the cohort. Patients who had inpatient coronary angiography were more likely to be male, younger and have fewer co-morbid conditions than those who did not (Table 2). Death within 1 year occurred in 30.6% patients who did not undergo inpatient coronary angiography compared with 5.7% in those that did. 72.8% of individuals with normal renal function (an eGFR>90 ml/minute/1.73 m^2^) underwent inpatient coronary angiography compared with 15.5% of those with an eGFR<30 ml/minute/1.73 m^2^ (Table 1).

**Table 2:** Selected covariates stratified by whether inpatient coronary angiography was performed or not, in 35 881 adults presenting with non-ST-elevation acute coronary syndrome (all data is presented as numbers with column percentage unless otherwise stated).

	IP Coronary angiography not performed	IP Coronary angiography performed
	N = 19 235	N = 16 646
**Demographic**		
Male gender	10 821 (56.3)	11 604 (69.7)
Age in years, median (IQR)	81 (72–87)	68 (56–76)
**Past Medical History**		
Hypertension	10 631 (55.3)	8 732 (52.5)
Stroke	2 842 (14.8)	1 146 (6.9)
PVD	1 228 (6.4)	7 42 (4.5)
Treated hyperlipidaemia	5 717 (29.7)	6 677 (40.1)
CCF	2 466 (12.8)	680 (4.1)
Previous MI	7 586 (39.4)	4 309 (26.4)
Previous PCI	1 559 (8.3)	2 231 (13.4)
Previous CABG	1 736 (9.0)	1 291 (7.8)
Diabetes Mellitus	5 030 (26.2)	3 530 (21.2)
Current smoker	2 912 (15.1)	46 22 (27.8)
**Diagnostics**		
Haemoglobin (g/dl), median (IQR)	12.6 (11.0–14.0)	14.0 (12.8–15.0)
Peak Troponin, median (IQR)	0.8 (0.2–3.7)	0.8 (0.2–3.6)
eGFR (ml/minute/1.73 m^2^)		
>90	1 762 (9.2)	4 720 (28.4)
60–90	6 274 (32.6)	7 445 (44.7)
45–59	4 377 (22.8)	2 613 (15.7)
30–44	4 086 (21.2)	1 366 (8.2)
15–29	2 249 (11.7)	416 (2.5)
<15	487 (2.5)	86 (0.5)
Systolic blood pressure (mmHg), mean (sd)	140 (29)	145 (27)
Heart rate (beats/min), median (IQR)	84 (70–100)	76 (65–90)

Abbreviations: IP = inpatient; IMD score = score of deprivation; PVD = peripheral vascular disease; CCF = congestive cardiac failure; MI = myocardial infarction; PCI = percutaneous coronary intervention; CABG = coronary artery bypass graft; eGFR = estimated glomerular filtration rate; IQR = interquartile range; sd = standard deviation.

After adjusting for all other comorbidities and covariables, there was a stepwise reduction in the odds of undergoing inpatient coronary angiography with increasing severity of renal impairment; a reduction of 33% in patients with eGFR 45–59 ml/minute/1.73 m^2^ (adjusted OR 0.67, 95% CI 0.55–0.81), 42% in those with an eGFR between 30–44 ml/minute/1.73 m^2^ (adjusted OR 0.58, 95% CI 0.48–0.70), and 64% in those with eGFR<30 ml/minute/1.73 m^2^ (adjusted OR 0.36, 95% CI 0.29–0.43) compared with patients with an eGFR>90 ml/minute/1.73 m^2^ (Table 3).

**Table 3:** Results of the multivariable logistic regression analysis in 35 881 individuals with non-ST-elevation acute coronary syndrome for the association between eGFR and inpatient coronary angiography.

eGFR(ml/minute/1.73 m^2^)	Age & genderadjusted OR(95% CI)	P-value(Wald)	MultivariableAdjusted OR(95% CI)*	P-value(Wald)
>90	1		1	
60–90	0.81 (0.71–0.93)	0.003	0.81 (0.70–0.94)	0.006
45–59	0.58 (0.48–0.70)	<0.001	0.67 (0.55–0.81)	<0.001
30–44	0.42 (0.35–0.51)	<0.001	0.58 (0.48–0.70)	<0.001
<30	0.21 (0.18–0.26)	<0.001	0.36 (0.29–0.43)	<0.001

*Multivariable model adjusted for age, ethnicity, gender, IMD score, systolic blood pressure, heart rate, haemoglobin, peak troponin, ECG diagnosis, history of angina, hyperlipidaemia, hypertension, peripheral vascular disease, cerebrovascular disease, chronic obstructive airways disease, congestive cardiac failure, previous percutaneous coronary intervention, previous coronary artery bypass graft, previous myocardial infarction, diabetes, current smoking status and hospital.

Abbreviations: OR = odds ratio; CI = confidence interval; eGFR = estimated glomerular filtration rate.

### Renal Impairment at the Time of Presentation with NSTE-ACS and the Association between Inpatient Coronary Angiography and Death within 1 Year

In patient coronary angiography was associated with a survival benefit in each eGFR category (Table 4). In those with an eGFR 60–90 ml/minute/1.73 m^2^ inpatient coronary angiography was associated with a reduction in the estimated odds of death of 70% (adjusted OR 0.29, 95% CI 0.25–0.33) and by 54% in those with an eGFR<30 ml/minute/1.73 m^2^ (adjusted OR 0.46, 95%CI 0.36–0.58). On restricting the analysis to a subgroup with improved balance in the distribution of baseline characteristics based on estimated propensity score (N = 16 617), the estimated survival benefit observed did not change, except in those with an eGFR<30 ml/minute/1.73 m^2^ in whom the estimated survival benefit was more conservative (adjusted OR 0.59, 95% CI 0.37–0.96) (data not shown).

**Table 4:** Results of the multivariable logistic regression analysis in 35 881 individuals with non-ST-elevation acute coronary syndrome for the association between inpatient coronary angiography and all-cause death.

eGFR(ml/minute/1·73 m^2^)	Inpatientangiography status	MultivariableAdjusted OR(95% CI)*	P-value(Wald)
>90	Inpatient angiography not performed	1	
	Inpatient angiography	0.21 (0.17–0.27)	<0.001
60–90	Inpatient angiography not performed	1	
	Inpatient angiography	0.29 (0.25–0.33)	<0.001
45–59	Inpatient angiography not performed	1	
	Inpatient angiography	0.37 (0.32–0.43)	<0.001
30–44	Inpatient angiography not performed	1	
	Inpatient angiography	0.41 (0.34–0.48)	<0.001
<30	Inpatient angiography not performed	1	
	Inpatient angiography	0.46 (0.36–0.58)	<0.001

*p-interaction (Wald test) between eGFR category and inpatient coronary angiography and mortality: <0.001.

*Multivariable model adjusted for age, ethnicity, gender, IMD score, systolic blood pressure, heart rate, haemoglobin, peak troponin, ECG diagnosis, history of angina, hyperlipidaemia, hypertension, peripheral vascular disease, cerebrovascular disease, chronic obstructive airways disease, congestive cardiac failure, previous percutaneous coronary intervention, previous coronary artery bypass graft, previous myocardial infarction, diabetes, current smoking status and hospital.

Abbreviations: OR = odds ratio; CI = confidence interval; eGFR = estimated glomerular filtration rate.

### Renal Impairment at the Time of Presentation with NSTE-ACS and the Association between Inpatient Revascularisation and Death within 1 Year

**Figure 2:**
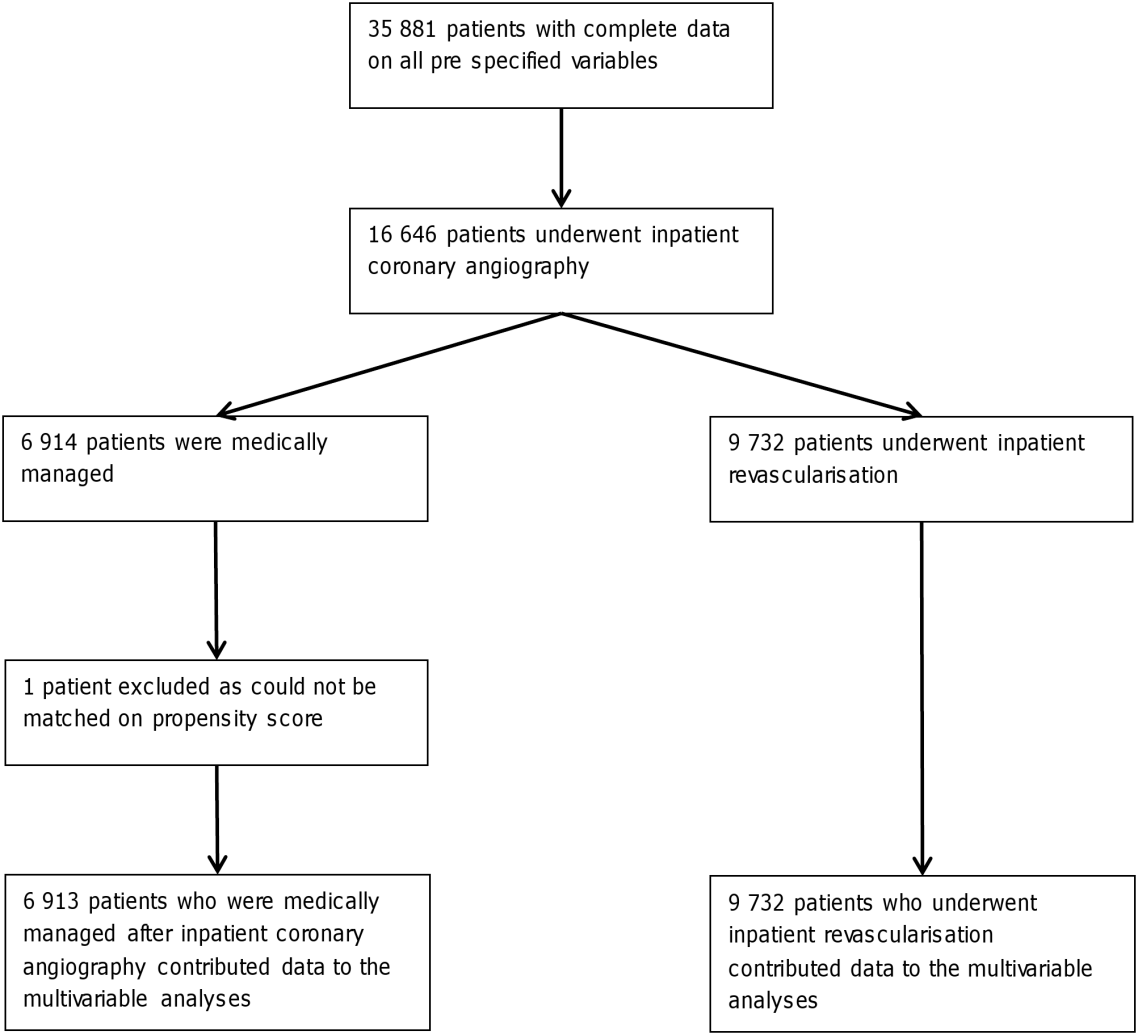
Number of patients in the complete case analysis contributing to various stages of the analysis.

Of 16 646 patients who had inpatient coronary angiography, 9 732 (58.5%) underwent inpatient revascularisation (Figure 2). On the basis of the propensity score, there was good overlap in the distribution of baseline characteristics between the group that underwent inpatient revascularisation and those who underwent angiography only (Appendix S1). Only 16% of patients with severe renal dysfunction at presentation (eGFR<30 ml/min/1.73 m^2^) underwent inpatient coronary angiography and could be considered for early revascularisation. However, of the 502 patients in this renal category that did have diagnostic angiography, nearly 50% underwent subsequent inpatient revascularisation (Table 5). The adjusted odds of undergoing inpatient revascularisation did not vary depending on eGFR category (data not shown).

**Table 5:** Selected covariates stratified by management strategy in 16 646 adults who underwent inpatient coronary angiography presenting with non-ST-elevation acute coronary syndrome.

	In patient MedicalManagement	In PatientRevascularisation
	N = 6 914	N = 9 732
**Demographic**		
Male gender	4 552 (65.8)	7 052 (72.5)
Age in years, median (IQR)	69 (60–77)	66 (57–75)
**Past Medical History**		
Hypertension	3 784 (54.7)	4 948 (50.8)
Stroke	563 (8.1)	583 (6.0)
PVD	359 (5.2)	383 (3.9)
Treated hyperlipidaemia	2 709 (39.2)	3 968 (40.8)
CCF	369 (5.3)	311 (3.2)
Previous MI	2 050 (29.7)	2 340 (24.0)
Previous PCI	912 (13.2)	1 319 (13.6)
Previous CABG	618 (8.9)	673 (6.9)
Diabetes Mellitus	1 594 (23.1)	1 936 (19.9)
Current smoker	1 656 (24.0)	2 966 (30.5)
**Diagnostics**		
Haemoglobin (g/dl), median (IQR)	13.8 (12.5–15.0)	14.0 (13.0–15.0)
Peak Troponin, median(IQR)	0.9 (0.2–4.0)	0.8 (0.2–3.3)
eGFR (ml/minute/1.73 m^2^)		
>90	1 728 (25.0)	2 992 (30.7)
60–90	3 023 (43.7)	4 422 (45.4)
45–59	1 243 (18.0)	1 370 (14.1)
30–44	669 (9.7)	697 (7.2)
15–29	211 (3.1)	205 (2.1)
<15	40 (0.6)	46 (0.5)
Systolic blood pressure (mmHg), mean (sd)	144 (27)	145 (28)
Heart rate (beats/min), median (IQR)	79 (67–93)	76 (65–88)

Abbreviations: PVD = peripheral vascular disease; CCF = congestive cardiac failure; MI = myocardial infarction; PCI = percutaneous coronary intervention; CABG = coronary artery bypass graft; eGFR = estimated glomerular filtration rate; IQR = interquartile range; sd = standard deviation.

All data is presented as numbers with column percentage unless otherwise stated. Where percentages do not equal 100% this is due to rounding.

538 deaths (7.8%) occurred within a year in those patients managed medically after inpatient coronary angiography compared with 413 deaths (4.2%) amongst patients who had inpatient revascularisation. After adjusting for co-variables, inpatient revascularisation was associated with a reduction in the odds of death within 1 year of approximately 30% (adjusted OR 0.66, 95%CI 0.57–0.77) (Table 6). When stratified by eGFR category there was a trend that the relative survival benefit of inpatient revascularisation may be less in those with an eGFR<30 ml/minute/1.73 m^2^ (adjusted OR 0.80, 95% CI 0.52–1.24) compared with the other eGFR categories (eGFR 60–90 ml/minute/1.73 m^2^ adjusted OR 0.63, 95% CI 0.49–0.81). However the confidence intervals between eGFR categories overlapped and there was no statistical evidence of modification by severity of renal dysfunction on the association between inpatient revascularisation and death (p-interaction = 0.744) (Table 6 and 7). There was weak evidence of effect modification by gender on this association with a trend to a lower adjusted odds of death in women (p-interaction = 0.060).

**Table 6:** Results of the adjusted logistic regression analysis in 16 645 individuals with non-ST-elevation acute coronary syndrome for the association between inpatient revascularisation and mortality compared with individuals who were medically managed after inpatient coronary angiography.

ManagementStrategy	Age & genderadjustedOR (95% CI)	P-value(Wald)	MultivariableAdjusted OR(95% CI)*	P-value(Wald)	Propensityscore adjustedOR (95% CI)	P-value(Wald)
Medical Mx	1		1		1	
In patient Revascularisation	0.60(0.52–0.70)	<0.001	0.66(0.57–0.77)	<0.001	0.68(0.58–0.80)	<0.001

*p-interaction (Wald test) between eGFR category and inpatient revascularisation and mortality: 0.744.

Multivariable Model adjusted for age, ethnicity, gender, IMD score, eGFR, systolic blood pressure, heart rate, haemoglobin, peak troponin, ECG diagnosis, history of angina, hyperlipidaemia, hypertension, peripheral vascular disease, cerebrovascular disease, chronic obstructive airways disease, congestive cardiac failure, previous percutaneous coronary intervention, previous coronary artery bypass graft, previous myocardial infarction, diabetes, current smoking status and hospital.

Propensity Score estimated using age, ethnicity, gender, IMD score, eGFR, systolic blood pressure, heart rate, haemoglobin, peak troponin ECG diagnosis, history of angina, hyperlipidaemia, hypertension, peripheral vascular disease, cerebrovascular disease, chronic obstructive airways disease, congestive cardiac failure, previous percutaneous coronary intervention, previous coronary artery bypass graft, previous myocardial infarction, diabetes, current smoking status.

Abbreviations: Medical Mx = medical management; OR = odds ratio; CI = confidence interval; eGFR = estimated glomerular filtration rate.

**Table 7:** Results of the adjusted logistic regression analysis in 16 645 individuals with non-ST-elevation acute coronary syndrome for the association between inpatient revascularisation and mortality compared with individuals who were medically managed after inpatient coronary angiography stratified by category of renal dysfunction.

eGFR(ml/minute/1.73 m^2^)	ManagementStrategy	MultivariableAdjusted OR(95% CI)*	P-value(Wald)
>90	Medical Mx	1	
	In patient Revascularisation	0.55(0.36–0.85)	0.008
60–90	Medical Mx	1	
	In patient Revascularisation	0.63(0.49–0.81)	<0.001
45–60	Medical Mx	1	
	In patient Revascularisation	0.69(0.51–0.95)	0.020
30–45	Medical Mx	1	
	In patient Revascularisation	0.68(0.49–0.94)	0.021
<30	Medical Mx	1	
	In patient Revascularisation	0.80(0.52–1.24)	0.320

*p-interaction (Wald test) between eGFR category and inpatient revascularisation and mortality: 0.744.

Multivariable Model adjusted for age, ethnicity, gender, IMD score, eGFR, systolic blood pressure, heart rate, haemoglobin, peak troponin, ECG diagnosis, history of angina, hyperlipidaemia, hypertension, peripheral vascular disease, cerebrovascular disease, chronic obstructive airways disease, congestive cardiac failure, previous percutaneous coronary intervention, previous coronary artery bypass graft, previous myocardial infarction, diabetes, current smoking status and hospital.

Propensity Score estimated using age, ethnicity, gender, IMD score, eGFR, systolic blood pressure, heart rate, haemoglobin, peak troponin ECG diagnosis, history of angina, hyperlipidaemia, hypertension, peripheral vascular disease, cerebrovascular disease, chronic obstructive airways disease, congestive cardiac failure, previous percutaneous coronary intervention, previous coronary artery bypass graft, previous myocardial infarction, diabetes, current smoking status.

Abbreviations: Medical Mx = medical management; OR = odds ratio; CI = confidence interval; eGFR = estimated glomerular filtration rate.

### Sensitivity Analysis

Results of the logistic regression model adjusted for the propensity score as a single co-variable demonstrated a similar reduction in the odds for death within 1 year associated with inpatient revascularisation (adjusted OR 0.68, 95% CI 0.58–0.80, Table 6).

Limiting the analysis to 5-day survivors did not alter the associations observed (Appendix S4a, 4b and 4c).

We repeated the analysis excluding 116 patients who had declined revascularisation. No change in the associations found in our main analysis was observed (data not shown).

Inclusion of aspirin, clopidogrel, ACE inhibitors, beta-blockers or statins prescribed at discharge in the model did not change the adjusted odds for death associated with inpatient revascularisation within 1 year (adjusted OR 0.68, 95% CI 0.57–0.80), with no evidence of modification by severity of renal dysfunction (p-interaction = 0.711).

After multiple imputation, the adjusted odds ratios were marginally more conservative (Appendix S5a and 5b; data not shown for the analysis of the association between inpatient coronary angiography and death).

## Discussion

In this study of over 35 000 individuals with NSTE-ACS in England and Wales, admitted to NHS hospitals between 2008 and 2010, we have demonstrated that renal dysfunction is common and that patients with renal impairment are much less likely to undergo inpatient diagnostic coronary angiography than patients with normal renal function. This association was maintained after adjusting for differences in numerous baseline characteristics and comorbidities and was observed across the range of renal impairment, including those patients with moderate renal dysfunction (eGFR 30–59 ml/minute/1.73 m^2^). Inpatient coronary angiography was associated with an improved survival. In those patients with moderate renal dysfunction that did undergo inpatient angiography, nearly 50% of patients then underwent revascularisation with a similar survival benefit as seen in patients with preserved renal function.

The majority of patients (84%) with severe renal dysfunction (eGFR<30 ml/min/1.73 m^2^) did not undergo inpatient diagnostic angiography. Therefore it is unclear whether, amongst this large group without inpatient diagnostic angiography, early revascularisation in those with suitable coronary lesions would have imparted a survival benefit.

Our finding that patients with renal dysfunction are less likely to undergo early coronary angiography than patients with preserved renal function is supported by several other analyses from different health care systems [1], [8]–[10], [25]. We suspect that the reasons are complex, reflecting both individual patient and clinician level factors as well as organisational factors spanning community and hospital level care. Patients with renal dysfunction are more likely to present with atypical clinical features [25], [26] and not necessarily directly to cardiologists. Clinical uncertainty as to the interpretation of troponin measurements in patients with renal impairment can compound diagnostic difficulties [27]. Concerns regarding the risk of acute kidney injury (AKI), in particular related to contrast-induced AKI, or a presumed increased risk of bleeding complications are also likely to influence management decisions [6], [28]. However, recent work suggests the risks of AKI associated with coronary angiography after ACS are overstated [9]. In addition, routine coronary angiography as part of renal transplant work-up in patients with advanced renal impairment is not associated with an accelerated decline in renal function [29]. As many patients with NSTE-ACS undergo angiography on a semi-urgent basis there are opportunities for clinicians to ensure adequate hydration and optimal angiographic practices that reduce the risk of AKI. In a previous study from the GRACE collaboration the most commonly reported reason for foregoing an early-invasive management strategy in those with renal impairment was insufficient risk (37.7%), while concerns over comorbidity (12.5%) and bleeding (7.2%) were minor in comparison [30]. However, the median GRACE score of those patients deemed ‘low risk’ was paradoxically high. Misrepresentation of risk and resultant denial of early-invasive management may contribute to worse outcomes in patients with renal dysfunction [30], and in other high risk groups in whom the same treatment paradox has been observed [31]–[33].

Earlier major clinical trials have compared a routine early invasive strategy with a selective invasive strategy after NSTE-ACS, rather than outcomes after revascularisation specifically [4], [34]–[36]. Patients with renal impairment have been under-represented in these studies [5] and no direct RCT evidence regarding an early invasive strategy, or specifically outcomes after revascularisation, are available in patients with renal impairment. A systematic review and meta-analysis of individual level data from five RCTs that had recorded information on renal function suggested that the benefits of an early invasive strategy are preserved in patients with renal impairment, with a trend in reduction of risk of death and non-fatal re-infarction at one year (in patients with chronic kidney disease (CKD) stages 3–5 i.e. an eGFR<60 ml/minute/1.73 m^2^, a pooled estimate risk ratio 0.76 (95% CI 0.49–1.17) was reported) [37]. Among the studies included in that meta-analysis the mean age ranged from 59–66 years, 14–28% had diabetes and mortality rates in the ‘conservative’ arms were 2.5–10%. Patients with CKD accounted for 19.4% (1 453/7 481) with the majority having an eGFR 30–60 ml/minute/1.73 m^2^ (80%). Our real world ACS cohort was quite different to those in the RCTs. In our study, the median age was 75 years, 40% had an eGFR at time of presentation of <60 ml/minute/1.73 m^2^ and at one year 30% of those who did not undergo inpatient coronary angiography had died.

Our analysis of the outcomes associated with inpatient coronary angiography will have included people in the comparison group (those that did not have inpatient coronary angiography) that would have been excluded from the randomised trials, and would therefore not have been considered for revascularisation, thus suggesting a possibly overoptimistic benefit of an early invasive approach. Some of the benefit observed in our comparison of those receiving and those not receiving inpatient angiography may also reflect other management differences between the groups, such as more aggressive antiplatelet or adjunctive medical therapies in the group in our cohort who underwent inpatient coronary angiography [1]. Thus, to further evaluate whether renal function modified outcomes after inpatient revascularisation we restricted the analysis to those in whom a clinical decision to consider revascularisation had been taken following inpatient coronary angiography.

Previous registry-based analyses have reported varied results. Data from the SWEDEHEART registry suggested that early revascularisation improved 1-year survival in patients with NSTE-ACS and mild-to-moderate renal insufficiency [8]. However, the observed benefit declined with lower renal function, and there was a trend toward harm in those with an eGFR<15 ml/minute/1.73 m^2^ or on dialysis (HR 1.61 95% CI 0.84–3.09). The wide confidence interval reflects the low number of patients in this eGFR category (n = 278, with 41 patients undergoing early revascularisation) making it hard to draw firm conclusions. In our study there was no statistical evidence of modification by eGFR category on the survival benefit associated with inpatient revascularisation, a finding supported by a study from the GRACE collaboration [30].

None of our analyses suggest that inpatient angiography or subsequent revascularisation was associated with harm in patients with renal dysfunction, though the fear of this may be influencing clinical judgement and decision making. Consistent with previous studies, the main barrier to revascularisation appears to be the decision to undertake inpatient coronary angiography [30]. The few patients with eGFR<30 ml/min/1.73 m^2^ that do undergo diagnostic angiography may represent a highly select subset of patients with severe renal impairment in whom an early invasive approach is likely to be of most benefit. However, the efficacy of a routine early invasive approach in individuals presenting with this severity of renal dysfunction currently remains essentially undefined.

Our cohort from a national ACS registry provides the most comprehensive account of current clinical practice in England and Wales in terms of the relationships between renal function, early angiography and revascularisation and patient outcomes after NSTE-ACS, and adds further contemporary data to the available research in this field. We have taken account of a range of confounders and have conducted multiple sensitivity analyses, including using datasets derived from multiple imputation, the results of which have supported the findings of our main analyses. However, there are limitations. Most importantly, this is an observational study and not a randomised controlled trial. Confounding may be present although we aimed to minimise this by incorporating a propensity score methodology and a wide range of baseline characteristics. We did not have a direct measure of true kidney function and used eGFR based on the CKD-EPI formula. As only a single creatinine was available for each patient we were unable to evaluate the components of chronic kidney disease or acute kidney injury. Our conclusions therefore refer to renal function at the time of presentation. However, given that historical creatinine values may not always be available to practising clinicians when they make decisions regarding angiography or revascularisation we argue that the findings of this analysis are relevant to clinical practice. Currently, identification of patients on dialysis or those with a renal transplant is not possible within the MINAP dataset. Very few individuals with an eGFR<30 ml/minute/1.73 m^2^ contributed to the analysis focussed on inpatient revascularisation and survival as so few had an inpatient coronary angiogram, so it is very likely that individuals on dialysis were excluded. We did not have details of coronary anatomy. After coronary angiography some patients will be treated medically because no treatable culprit lesion is present and others because revascularisation carries unacceptable risk or is unlikely to be successful. Having this information would enable a much more detailed description of the differences between patients with various degrees of renal function, and a deeper understanding of management strategies used and patient outcomes. We lacked information on other important characteristics that stratify risk (in patients who are not offered a routine invasive approach), for example the results of stress tests and measurements of left ventricular function. Nor did we have information on clinical events in hospital, such as further myocardial infarction, which may have influenced clinical decision making. While we were able to categorise patients into those with and those without angiography (and subsequent revascularisation) we lacked information regarding delay from admission to intervention. To evaluate the risk of potential survivor bias we undertook sensitivity analyses restricted to those who survived more than five days, but this is an important limitation. Other outcomes such as cardiac specific mortality, in-hospital mortality and length of stay would be valuable additional information. As mentioned above, our analysis may also have lacked power to detect evidence of modification by category of renal dysfunction on outcomes by management strategy due to the relatively low numbers of individuals with severe renal impairment contributing to that analysis.

Our analyses from MINAP provide further evidence that patients with renal dysfunction are much less likely to undergo inpatient coronary angiography than individuals with preserved renal function which is not explained by associated comorbidity. Inpatient coronary angiography was associated with improved survival across all categories of renal dysfunction. After inpatient angiography, relative outcomes following revascularisation were not modified by severity of renal dysfunction. As in previous studies however low patient numbers with severe renal dysfunction limit the ability to draw firm conclusions.

There is a discrepancy between the care advised in clinical guidelines regarding an early invasive strategy in patients with renal dysfunction and NSTE-ACS, and care delivered in clinical practice. Further research is required to understand why this variation exists and determine whether there are missed opportunities for quality improvement. In patients with severe renal impairment or those on dialysis a RCT is required to definitively evaluate the efficacy and optimal timing of early angiography and subsequent revascularisation after NSTE-ACS.

## Supporting Information

Appendix S1 Distribution of the conditional propensity scores for undergoing inpatient revascularisation after inpatient coronary angiography.
(TIF)

Appendix S2 Frequency of missing data in the non-ST-elevation acute coronary syndrome dataset.
(DOCX)

Appendix S3 Key covariates in the patients included in the complete case analysis and patients that were excluded due to incomplete data.
(DOCX)

Appendix S4 Comparison between the results of the complete case analysis and the analysis restricted to patients who survived for 5 days or more.
(DOCX)

Appendix S5 Comparison between the results of the complete case analysis and the analysis using 10 datasets derived using multiple imputation.
(DOCX)
