# Supplementary figures and images for: Inpatient Coronary Angiography and Revascularisation following Non-ST-Elevation Acute Coronary Syndrome in Patients with Renal Impairment: A Cohort Study Using the Myocardial Ischaemia National Audit Project
Source: PLoS One. 2014 Jun 17;9(6):e99925. doi: 10.1371/journal.pone.0099925 (PMC4061061; doi:10.1371/journal.pone.0099925)

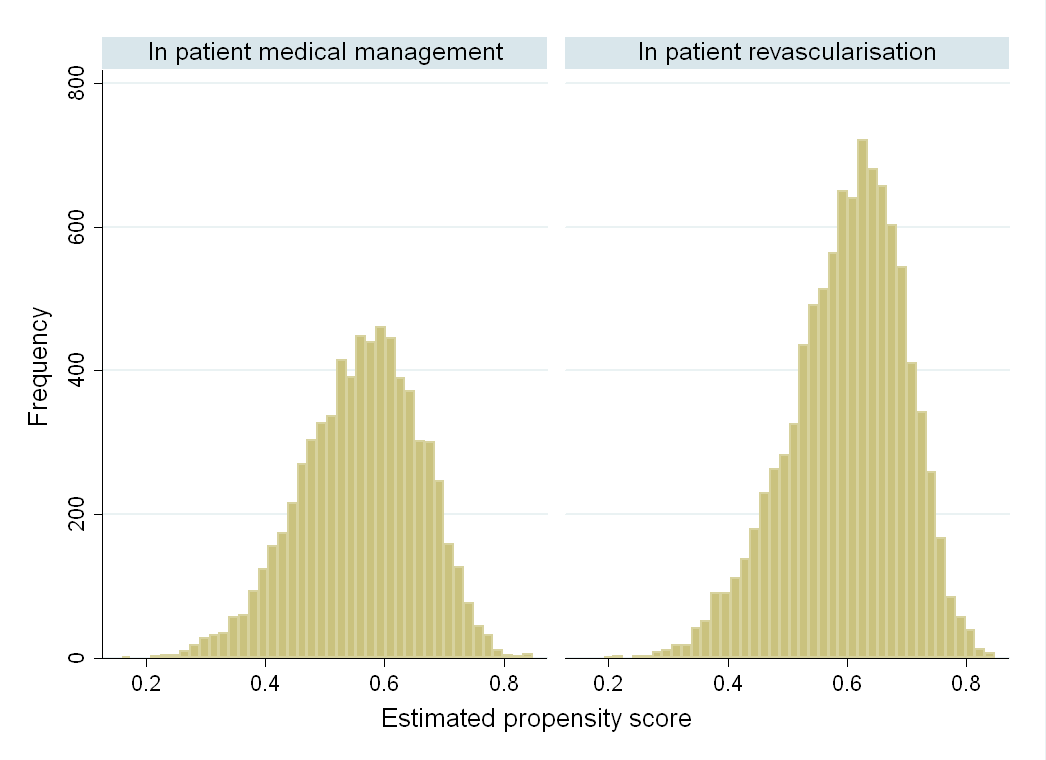

Supplement: Appendix S1 — Distribution of the conditional propensity scores for undergoing inpatient revascularisation after inpatient coronary angiography. (TIF) [file pone.0099925.s001.tif]
