# Supplementary material for: Inpatient Coronary Angiography and Revascularisation following Non-ST-Elevation Acute Coronary Syndrome in Patients with Renal Impairment: A Cohort Study Using the Myocardial Ischaemia National Audit Project
Source: PLoS One. 2014 Jun 17;9(6):e99925. doi: 10.1371/journal.pone.0099925 (PMC4061061; doi:10.1371/journal.pone.0099925)
Supplement: Appendix S2 — Frequency of missing data in the non-ST-elevation acute coronary syndrome dataset. (DOCX) [file pone.0099925.s002.docx]

Appendix S2. Frequency of missing data in the non-ST-elevation acute coronary syndrome dataset

(all data is presented as numbers with column percentage unless otherwise stated)

| **Variable** | NSTE-ACS dataset  N=91 342 |
| --- | --- |
| **Demographic** |  |
| Sex | 111 (0.1) |
| Age in years, median (IQR) | 25 (0.03) |
| Ethnicity | 8 966 (9.8) |
| IMD Score | 7 691 (8.4) |
| Seen by cardiology | 8 584 (9.4) |
| **Past Medical History** |  |
| Hypertension | 4 876 (5.3) |
| Previous angina | 5 567 (6.1) |
| Stroke | 6 763 (7.4) |
| PVD | 9 622 (10.5) |
| Treated hyperlipidaemia | 7 013 (7.7) |
| CCF | 7 069 (7.7) |
| Previous MI | 4 682 (5.1) |
| Previous PCI | 6 133 (6.7) |
| Previous CABG | 5 815 (6.4) |
| Current smoker | 7 516 (8.2) |
| Diabetes Mellitus | 1 792 (2.0) |
| COPD | 8 246 (9.0) |
| **Diagnostics** |  |
| ECG appearance | 6 256 (6.9) |
| Haemoglobin in g/dl, | 17 032 (18.6) |
| Peak troponin | 1 960 (2.1) |
| Creatinine in µmol/L) | 11 256 (12.3) |
| eGFR ml/minute/1.73m^2^ | 16 632 (18.2) |
| Heart rate in beats/min | 11 372 (12.4) |
| Systolic blood pressure in mmHg | 11 084 (12.1) |
| Coronary angiography | 4 155 (4.5) |
| Inpatient revascularisation | 16 751 (18.3) |
| Death | 336 (0.4) |

Abbreviations: IMD score=score of deprivation; PVD= peripheral vascular disease; CCF= congestive cardiac failure; MI= myocardial infarction; PCI= percutaneous coronary intervention; CABG= coronary artery bypass graft; COPD= chronic obstructive airways disease; ECG= electrocardiogram; eGFR= estimated glomerular filtration rate; IQR=interquartile range; SD=standard deviation
