# Supplementary material for: Inpatient Coronary Angiography and Revascularisation following Non-ST-Elevation Acute Coronary Syndrome in Patients with Renal Impairment: A Cohort Study Using the Myocardial Ischaemia National Audit Project
Source: PLoS One. 2014 Jun 17;9(6):e99925. doi: 10.1371/journal.pone.0099925 (PMC4061061; doi:10.1371/journal.pone.0099925)
Supplement: Appendix S3 — Key covariates in the patients included in the complete case analysis and patients that were excluded due to incomplete data. (DOCX) [file pone.0099925.s003.docx]

Appendix S3. Key covariates in the patients included in the complete case analysis and patients that were excluded due to incomplete data (all data is presented as numbers with column percentage unless otherwise stated)

|  | **Not included in complete case analysis** | **Included in complete case analysis** |
| --- | --- | --- |
| **Demographic** |  |  |
| Gender, N | 55 350 | 35 881 |
| Male, N(%) | 34 588 (62.5) | 22 425 (62.5) |
| Age, N | 55 436 | 35 881 |
| Age in years, median (IQR) | 74 (63-82) | 75 (64-83) |
| Ethnicity, N | 46 495 | 35 881 |
| White, N(%) | 43 877 (94.4) | 33 920 (94.5) |
| Black, N(%) | 249 (0.5) | 217 (0.6) |
| Asian/Other, N(%) | 2 369 (5.1) | 1 744 (4.9) |
| IMD Score, N | 47 770 | 35 881 |
| Median IMD score(IQR) | 16.6 (9.2-29.6) | 17.4 (9.7-30.5) |
| **Past Medical History** |  |  |
| Hypertension, N | 50 585 | 35 881 |
| Yes, N(%) | 28 120 (55.6) | 19 363 (54.0) |
| Previous angina, N | 49 894 | 35 881 |
| Yes, N(%) | 18 567 (37.2) | 13 804 (38.5) |
| Stroke, N | 48 698 | 35 881 |
| Yes, N(%) | 5 097 (10.5) | 3 988 (11.1) |
| PVD, N | 45 839 | 35 881 |
| Yes, N(%) | 2 662 (5.8) | 1 970 (5.5) |
| Treated lipids, N | 48 448 | 35 881 |
| Yes, N(%) | 18 629 (38.5) | 12 394 (34.5) |
| CCF, N | 48 392 | 35 881 |
| Yes, N(%) | 4 127 (8.5) | 3 146 (8.8) |
| Previous MI, N | 50 779 | 35 881 |
| Yes, N(%) | 16 739 (33.0) | 11 976 (33.4) |
| Previous PCI, N | 49 328 | 35 881 |
| Yes, N(%) | 5 900 (12.0) | 3 830 (10.7) |
| Previous CABG, N | 49 646 | 35 881 |
| Yes, N(%) | 4 595 (9.3) | 3 027 (8.4) |
| Current smoker, N | 47 945 | 35 881 |
| Yes, N(%) | 10 704 (22.3) | 7 534 (21.0) |
| Diabetes Mellitus, N | 53 669 | 35 881 |
| Yes, N(%) | 12 814 (23.9) | 8 560 (23.9) |
| COPD, N | 47 215 | 35 881 |
| Yes, N(%) | 8 127 (17.2) | 6 100 (17.0) |
|  |  |  |
|  |  |  |
|  |  |  |
|  | **Not included in complete case analysis** | **Included in complete case analysis** |
| **Diagnostics** |  |  |
| ECG appearance, N | 49 205 | 35 881 |
| Normal ECG, N(%) | 6 934 (14.1) | 4 685 (13.1) |
| LBBB, N(%) | 3 624 (7.4) | 2 743 (7.6) |
| ST segment depression, N(%) | 13 610 (27.7) | 10 269 (28.6) |
| T wave changes, N(%) | 13 031 (26.5) | 9 650 (26.9) |
| Other ECG abnormality, N(%) | 12 006 (24.4) | 8 534 (23.8) |
| Haemoglobin, N | 38 429 | 35 881 |
| Haemoglobin in g/dl, median (IQR) | 13.3 (12.0-14.7) | 13.2 (11.8-14.6) |
| Peak troponin | 53 501 | 35 881 |
| Peak troponin, median (IQR) | 0.7 (0.2-3.3) | 0.8 (0.2-3.7) |
| eGFR in ml/minute/1.73m^2^, N | 38 829 | 35 881 |
| >90, N(%) | 7 761 (20.0) | 6 482 (18.1) |
| 60-90, N(%) | 14 268 (36.8) | 13 719 (38.2) |
| 45-59, N(%) | 7 255 (18.7) | 6 990 (19.5) |
| 30-44, N(%) | 5 874 (15.1) | 5 452 (15.2) |
| 15-29, N(%) | 2 905 (7.5) | 2 665 (7.4) |
| <15, N(%) | 766 (2.0) | 573 (1.6) |
| Heart rate, N | 44 089 | 35 881 |
| Heart rate in beats/min, median (IQR) | 80 (68-96) | 80 (68-96) |
| Systolic blood pressure, N | 44 377 | 35 881 |
| Systolic blood pressure in mmHg, mean (sd) | 141 (29) | 142 (29) |
| Coronary angiography, N | 51 306 | 35 881 |
| Yes, N(%) | 28 693 (55.9) | 16 646 (46.9) |
| Inpatient revascularisation, N | 38 710 | 35 881 |
| Yes, N(%) | 11 721 (30.3) | 9 732 (27.1) |
| Death, N | 55 125 | 35 881 |
| Death, Y(%) | 8 721 (15.8) | 6 831 (19.0) |

*as there is some missing data within each variable total numbers with data available for each variable are shown.

Abbreviations: IMD score=score of deprivation; PVD= peripheral vascular disease; CCF= congestive cardiac failure; MI= myocardial infarction; PCI= percutaneous coronary intervention; CABG= coronary artery bypass graft; COPD= chronic obstructive airways disease; ECG= electrocardiogram; LBBB= left bundle branch block; eGFR= estimated glomerular filtration rate; IQR=interquartile range; sd=standard deviation; N=number of patient
