# Supplementary material for: Inpatient Coronary Angiography and Revascularisation following Non-ST-Elevation Acute Coronary Syndrome in Patients with Renal Impairment: A Cohort Study Using the Myocardial Ischaemia National Audit Project
Source: PLoS One. 2014 Jun 17;9(6):e99925. doi: 10.1371/journal.pone.0099925 (PMC4061061; doi:10.1371/journal.pone.0099925)
Supplement: Appendix S4 — Comparison between the results of the complete case analysis and the analysis restricted to patients who survived for 5 days or more. (DOCX) [file pone.0099925.s004.docx]

Appendix S4a.

Comparison between the results of the complete case analysis and the analysis restricted to patients who survived for 5 days or more: the association between eGFR and inpatient coronary angiography in patients with non-ST-elevation acute coronary syndrome

| eGFR (ml/minute/1.73m^2^) | Multivariable Adjusted  OR- complete case analysis  (95% CI) | P-value  (Wald) | Multivariable Adjusted  OR- 5 day survivors  (95% CI) ** | P-value  (Wald) |
| --- | --- | --- | --- | --- |
| >90 | 1 |  | 1 |  |
| 60-90 | 0.81 (0.70-0.94) | 0.006 | 0.81 (0.70-0.94) | 0.006 |
| 45-59 | 0.67 (0.55-0.81) | <0.001 | 0.67 (0.56-0.82) | 0.001 |
| 30-44 | 0.58 (0.48-0.70) | <0.001 | 0.59 (0.49-0.72) | <0.001 |
| <30 | 0.36 (0.29-0.43) | <0.001 | 0.37(0.30-0.46) | <0.001 |

*Multivariable Model adjusted for age, ethnicity, gender, IMD score, systolic blood pressure, heart rate, haemoglobin, peak troponin, ECG diagnosis, history of angina, hyperlipidaemia, hypertension, peripheral vascular disease, cerebrovascular disease, chronic obstructive airways disease, congestive cardiac failure, previous percutaneous coronary intervention, previous coronary artery bypass graft, previous myocardial infarction, diabetes, current smoking status and hospital

Abbreviations: OR=odds ratio; CI=confidence interval; eGFR= estimated glomerular filtration rate

Appendix S4b.Comparison between the results of the complete case analysis and the analysis restricted to patients who survived for 5 days or more: results of the multivariable logistic regression analysis in individuals with non-ST-elevation acute coronary syndrome for the association between inpatient coronary angiography and all-cause death:

| eGFR  (ml/minute/1·73m^2^) | Inpatient angiography status | Multivariable Adjusted  OR  (95% CI) * | P-value  (Wald) | Multivariable Adjusted  OR-5 day survivors  (95% CI) * | P-value  (Wald) |  |
| --- | --- | --- | --- | --- | --- | --- |
| >90 | Inpatient angiography not performed | 1 |  | 1 |  |  |
|  | Inpatient angiography | 0.21 (0.17-0.27) | <0.001 | 0.22 (0.18-0.29) | <0.001 |  |
| 60-90 | Inpatient angiography not performed | 1 |  | 1 |  |  |
|  | Inpatient angiography | 0.29 (0.25-0.33) | <0.001 | 0.30 (0.26-0.36) | <0.001 |  |
| 45-59 | Inpatient angiography not performed | 1 |  | 1 |  |  |
|  | Inpatient angiography | 0.37 (0.32-0.43) | <0.001 | 0.41 (0.36-0.47) | <0.001 |  |
| 30-44 | Inpatient angiography not performed | 1 |  | 1 |  |  |
|  | Inpatient angiography | 0.41 (0.34-0.48) | <0.001 | 0.46 (0.39-0.55) | <0.001 |  |
| <30 | Inpatient angiography not performed | 1 |  | 1 |  |  |
|  | Inpatient angiography | 0.46 (0.36-0.58) | <0.001 | 0.53 (0.41-0.67) | <0.001 |  |

*p-interaction (Wald test) between eGFR category and inpatient coronary angiography and mortality: <0.001 *Multivariable model adjusted for age, ethnicity, gender, IMD score, systolic blood pressure, heart rate, haemoglobin, peak troponin, ECG diagnosis, history of angina, hyperlipidaemia, hypertension, peripheral vascular disease, cerebrovascular disease, chronic obstructive airways disease, congestive cardiac failure, previous percutaneous coronary intervention, previous coronary artery bypass graft, previous myocardial infarction, diabetes, current smoking status and hospital.

Abbreviations: OR=odds ratio; CI=confidence interval; eGFR= estimated glomerular filtration rate

Appendix S4c.

Comparison between the results of the complete case analysis and the analysis restricted to patients who survived for 5 days or more: the association between inpatient revascularisation and mortality, compared with medical management after inpatient coronary angiography in patients with non-ST-elevation acute coronary syndrome

| Management  Strategy | Multivariable Adjusted  OR complete case analysis  (95% CI) * | P-value  (Wald) | Multivariable Adjusted  OR – 5 day survivors  (95% CI) ** | P-value  (Wald) |
| --- | --- | --- | --- | --- |
| Medical Mx | 1 |  | 1 |  |
| In patient Revascularisation | 0.66  (0.57-0.77) | <0.001 | 0.66  (0.56-0.77) | < 0.001 |

*p-interaction (Wald test) between eGFR category and inpatient revascularisation and mortality=0.744

**p-interaction (Wald test) between eGFR category and inpatient revascularisation and mortality=0.751

Model adjusted for age, ethnicity, gender, IMD score, eGFR systolic blood pressure, heart rate, haemoglobin, peak troponin, ECG diagnosis, history of angina, hyperlipidaemia, hypertension, peripheral vascular disease, cerebrovascular disease, chronic obstructive airways disease, congestive cardiac failure, previous percutaneous coronary intervention, previous coronary artery bypass graft, previous myocardial infarction, diabetes, current smoking status and hospital

Abbreviations: Medical Mx=medical management; IP revascularisation=in patient revascularisation; OR=odds ratio; CI=confidence interval; eGFR= estimated glomerular filtration rate
