# Supplementary material for: Inpatient Coronary Angiography and Revascularisation following Non-ST-Elevation Acute Coronary Syndrome in Patients with Renal Impairment: A Cohort Study Using the Myocardial Ischaemia National Audit Project
Source: PLoS One. 2014 Jun 17;9(6):e99925. doi: 10.1371/journal.pone.0099925 (PMC4061061; doi:10.1371/journal.pone.0099925)
Supplement: Appendix S5 — Comparison between the results of the complete case analysis and the analysis using 10 datasets derived using multiple imputation. (DOCX) [file pone.0099925.s005.docx]

Appendix S5a.

Comparison between the results of the complete case analysis and the analysis using 10 datasets derived using multiple imputation: the association between eGFR and inpatient coronary angiography in patients with non-ST-elevation acute coronary syndrome

| eGFR (ml/minute/1.73m^2^) | Multivariable Adjusted  OR- complete case analysis  (95% CI) | P-value  (Wald) | Multivariable Adjusted  OR- multiple imputation  (95% CI) ** | P-value  (Wald) |
| --- | --- | --- | --- | --- |
| >90 | 1 |  | 1 |  |
| 60-90 | 0.82 (0.71-0.94) | 0.126 | 0.89 (0.79-1.00) | 0.054 |
| 45-59 | 0.67 (0.55-0.81) | 0.003 | 0.76 (0.65-0.89) | 0.001 |
| 30-44 | 0.58 (0.48-0.70) | <0.001 | 0.63 (0.54-0.73) | <0.001 |
| 15-29 | 0.38 (0.31-0.47) | <0.001 | 0.39 (0.33-0.47) | <0.001 |
| <15 | 0.26 (0.19-0.35) | <0.001 | 0.31 (0.26-0.38) | <0.001 |

*Multivariable Model adjusted for age, ethnicity, gender, IMD score, systolic blood pressure, heart rate, haemoglobin, peak troponin, ECG diagnosis, history of angina, hyperlipidaemia, hypertension, peripheral vascular disease, cerebrovascular disease, chronic obstructive airways disease, congestive cardiac failure, previous percutaneous coronary intervention, previous coronary artery bypass graft, previous myocardial infarction, diabetes, current smoking status and hospital

Abbreviations: OR=odds ratio; CI=confidence interval; eGFR= estimated glomerular filtration rate

Appendix S5b.

Comparison between the results of the complete case analysis and the analysis using 10 datasets derived using multiple imputation: the association between inpatient revascularisation and mortality, compared with medical management after inpatient coronary angiography in patients with non-ST-elevation acute coronary syndrome

| Management  Strategy | Multivariable Adjusted  OR- complete case analysis  (95% CI) * | P-value  (Wald) | Multivariable Adjusted  OR- multiple imputation  (95% CI) ** | P-value  (Wald) |
| --- | --- | --- | --- | --- |
| Medical Mx | 1 |  | 1 |  |
| In patient Revascularisation | 0.66  (0.57-0.77) | <0.001 | 0.72  (0.65-0.80) | < 0.001 |

*p-interaction (Wald test) between eGFR category and inpatient revascularisation and mortality: 0.744

**p-interaction (Wald test) between eGFR category and inpatient revascularisation and mortality: 0.588

Multivariable Model adjusted for age, ethnicity, gender, IMD score, eGFR systolic blood pressure, heart rate, haemoglobin, peak troponin, ECG diagnosis, history of angina, hyperlipidaemia, hypertension, peripheral vascular disease, cerebrovascular disease, chronic obstructive airways disease, congestive cardiac failure, previous percutaneous coronary intervention, previous coronary artery bypass graft, previous myocardial infarction, diabetes, current smoking status and hospital

Abbreviations: Medical Mx=medical management; IP revascularisation=in patient revascularisation; OR=odds ratio; CI=confidence interval; eGFR= estimated glomerular filtration rate
